# Clinical Utility of FDG PET/CT in Patients with Autoimmune Pancreatitis: a Case-Control Study

**DOI:** 10.1038/s41598-018-21996-5

**Published:** 2018-02-26

**Authors:** Mei-Fang Cheng, Yue Leon Guo, Ruoh-Fang Yen, Yi-Chieh Chen, Chi-Lun Ko, Yu-Wen Tien, Wei-Chih Liao, Chia-Ju Liu, Yen-Wen Wu, Hsiu-Po Wang

**Affiliations:** 1 Department of Nuclear Medicine, National Taiwan University College of Medicine and Hospital, Taipei City, Taiwan; 2 Institute of Occupational Medicine and Industrial Hygiene, National Taiwan University, Taipei, Taiwan; 3 Environmental and Occupational Medicine, National Taiwan University College of Medicine and Hospital, Taipei City, Taiwan; 4 Department of Nuclear Medicine, National Taiwan University Hospital, Yun-Lin Branch, Yun-Lin County, Taiwan; 5 Department of Surgery, National Taiwan University College of Medicine and Hospital, Taipei City, Taiwan; 6 Department of Nuclear Medicine and Cardiovascular Medical Center (Cardiology), Far Eastern Memorial Hospital, New Taipei City, Taiwan; 7 National Yang-Ming University School of Medicine, Taipei, Taiwan; 8 Department of Internal Medicine, National Taiwan University College of Medicine and Hospital, Taipei City, Taiwan

## Abstract

Autoimmune pancreatitis (AIP) shares overlapping clinical features with pancreatic cancer (PC). Importantly, treatment of the two conditions is different. We investigated the clinical usefulness of ^18^F-fluorodeoxyglucose (FDG) positron emission tomography/computed tomography (PET/CT) in patients with suspected AIP before treatment. From September 2008 to July 2016, 53 patients with suspected AIP at National Taiwan University Hospital had PET/CT prior to therapy to exclude malignancy and evaluate the extent of inflammation. Their scans were compared with those from 61 PC patients. PET imaging features were analyzed using logistic regression. Significant differences in pancreatic tumor uptake morphology, maximum standardized uptake value, high-order primary tumor texture feature (i.e. high-gray level zone emphasis value), and numbers and location of extrapancreatic foci were found between AIP and PC. Using the prediction model, the area under curve of receiver-operator curve was 0.95 (*P* < 0.0001) with sensitivity, specificity, positive predictive, and negative predictive values of 90.6%, 84.0%, 87.9%, and 87.5% respectively, in differentiating AIP from PC. FDG PET/CT offers high sensitivity, albeit slightly lower specificity in differentiating AIP from PC. Nonetheless, additional systemic inflammatory foci detected by the whole body PET/CT help confirm diagnosis of AIP in these patients before initiating steroid therapy, especially when biopsy is inconclusive.

## Introduction

Positron emission tomography and computed tomography (PET/CT) using ^18^F-fluorodeoxyglucose (FDG) has been used in pancreatic cancer (PC) to survey distant metastasis before treatment and to evaluate therapeutic response^1,2^. Lymphoplasmacytic sclerosing pancreatitis has been unexpectedly identified in pathological specimens of patients initially diagnosed with PC, which was later confirmed to be autoimmune pancreatitis (AIP). AIP is a unique form of pancreatitis recognized in recent years as part of systemic immunoglobulin G4-related disease (IgG4-RD)^3^. Due to its similar clinical presentation, PC can be difficult to differentiate from AIP clinically. Although characteristic features of “sausage-like” enlargement of the pancreas with a “capsule-like” rim accompanied by narrowing of the main pancreatic duct in contrast-enhanced CT are diagnostic of AIP, many patients present with atypical imaging features making it difficult to differentiate from PC^4^. This is especially true in patients exhibiting a focal pancreatic mass and dilated pancreatic duct^4^. Even though an elevated serum IgG4 level can be present in type I AIP patients, type II AIP patients have normal serum IgG4 level, and some PC patients show elevated titers^5,6^. Biopsy of the pancreatic mass is can be non-diagnostic, especially with insufficient histological samples. Importantly, treatment of the two conditions is different: patients with PC should receive prompt surgery, whereas steroid therapy is the first line treatment for AIP. Moreover, patients with AIP have been reported to have a higher incidence of co-existing malignancy.

Therefore, this study aimed to determine whether FDG PET/CT could provide clinical useful information in patients with suspected AIP before initiation of steroid therapy.

## Results

**Figure 1:**
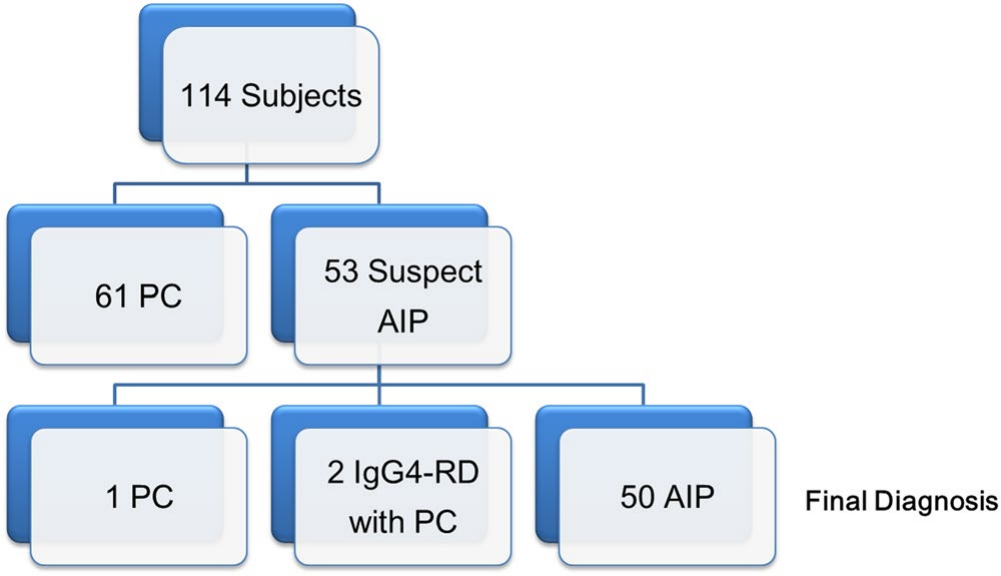
Final results of the 114 study patients. AIP, autoimmune disease; IgG4-RD, Immunoglobulin G4-related autoimmune disease; PC, pancreatic cancer.

FDG PET/CT confirmed systemic inflammatory lesions in 52 of 53 patients with suspected AIP, in which 2 had IgG4-RD but not AIP. Pancreatic malignancies were subsequently proved in these 3 patients (Fig. 1). All diagnosed AIP patients (a total of 50) fit the 2011 International Consensus Diagnostic Criteria for AIP^7^ at the time of this analysis. Among the 50 patients confirmed of AIP after PET scan, 11 (22.0%) had histopathological features of lymphoplasmacytic sclerosing pancreatitis with >10 IgG4-positive plasma cells per high power field and an IgG4:IgG cell ratio of at least 40%^7^. Thus, 11 subjects were of definitive type I AIP, 37 probable type I AIP, 1 probable type II AIP, and 1 AIP-not otherwise specified. In the 64 PC patients (including 3 patients initially diagnosed of AIP), reference standards were the surgical specimens in 52 (81.3%), biopsies guided by ultrasonography in 11 (17.2%), and CT-guided biopsy in 1 (1.6%). All histopathological confirmations were made within 1 month of FDG PET/CT. At the time of the analysis, 29 of the 114 (25.4%) patients died, all of which were from the PC group. Ulcerative colitis was incidentally found in FDG PET/CT in 2 AIP patients. Two other AIP patients had hepatocellular carcinoma and laryngeal cancer more than 5 years after their initial FDG PET/CT scan. None of the AIP patients developed pancreatic malignancy during the follow-up period and were all regularly followed up at the outpatient clinic at the time of this analysis (median, 36.9 months; range, 6–82.9 months).

The ranges of the examined PET parameters are shown in the Table from Supplementary data. Mean MTV was 34.4 ± 5.6 mL for PC, and 49.1 ± 5.3 mL for AIP. Three patients (3/64, 4.7%) from PC group had diffuse pancreatic uptake, and the remaining 58 (58/64, 90.6%) had localized pancreatic morphology. AIP patients were nearly equally distributed with respect to localized and diffuse pancreatic uptake morphology. Forty-one of 50 (82.0%) confirmed AIP patients had at least one site of extrapancreatic inflammation. In contrast to 16 (16/64, 25.0%) of the PC patients, half (25/50) of AIP patients had more than 2 sites. AIP and PC patients also differed with respect to sites of extrapancreatic lesions (Table 2). Extra-abdominal lymph nodes, mostly the mediastinum (25/50, 50.0%) and salivary glands (26/50, 52.0%) were the most frequent extrapancreatic lesions in AIP patients; whereas, liver (13/64, 20.3%) and abdominal lymph nodes (9/64, 14.1%) were more frequently seen in PC patients. Moreover, some sites of extrapancreatic uptake were only observed in the AIP group: lacrimal glands (1/50, 2.0%), axillary lymph nodes (7/50, 14.0%), vessel walls (8/50, 16%), and the pituitary gland (3/50, 6.0%).

**Table 1:** Clinical Features of the Participants at the Time of PET/CT (*n* = 114).

	Suspected AIP (*n* = 53)	Pancreatic Cancer (*n* = 61)
Age (*years*)	63.0 ± 14.0	65.0 ± 15.0
Males/females*, *n*	47/6	33/28
Body mass index (kg/m^2^)	22.0 ± 2.8	24.0 ± 2.7
Diabetes*, *n*	27	20
Serum IgG4 > 135 mg/dL*, *n*	50	4
Serum CA 19-9 level >37 U/mL*, *n*	16	43

Note: data are mean ± SD; *Significantly different between the two groups by student’s t-test or Chi-square test; IgG4-RD, Immunoglobulin G4-related autoimmune disease; IgG, Immunoglobulin; SD, standard deviation; N/A, Not applicable.

**Table 2:** Site of Extrapancreatic Lesions in 114 Patients.

Site	Pancreatic cancer (*n* = 64)	Autoimmune pancreatitis (*n* = 50)
Lacrimal glands	0 (0.0)	1 (2)
Salivary glands	4 (6.3)	26 (52)
Extra-abdominal LAPs		
Supraclavicular	1 (1.6)	3 (6)
Axillary	0 (0.0)	7 (14)
Mediastinal	7 (10.9)	25 (50)
Abdominal LAPs	9 (14.1)	2 (4)
Lung	5 (7.8)	0 (0)
Biliary tract	1 (1.6)	5 (10)
Liver	13 (20.3)	1 (2)
Retroperitoneum (including kidneys)	1 (1.6)	8 (16)
Vessels	0 (0.0)	8 (16)
Pituitary gland	0 (0.0)	3 (6)
Bone	3 (4.7)	0 (0)

Note: data in parentheses are percentages; LAPs: lymphadenopathy.

### Univariate Analysis

Eleven morphological parameters were statistically significant (*P* < 0.01) in differentiating AIP from PC according to univariate analysis (Table from Supplementary data).

### Backward Stepwise Logistic Regression Analysis

**Figure 2:**
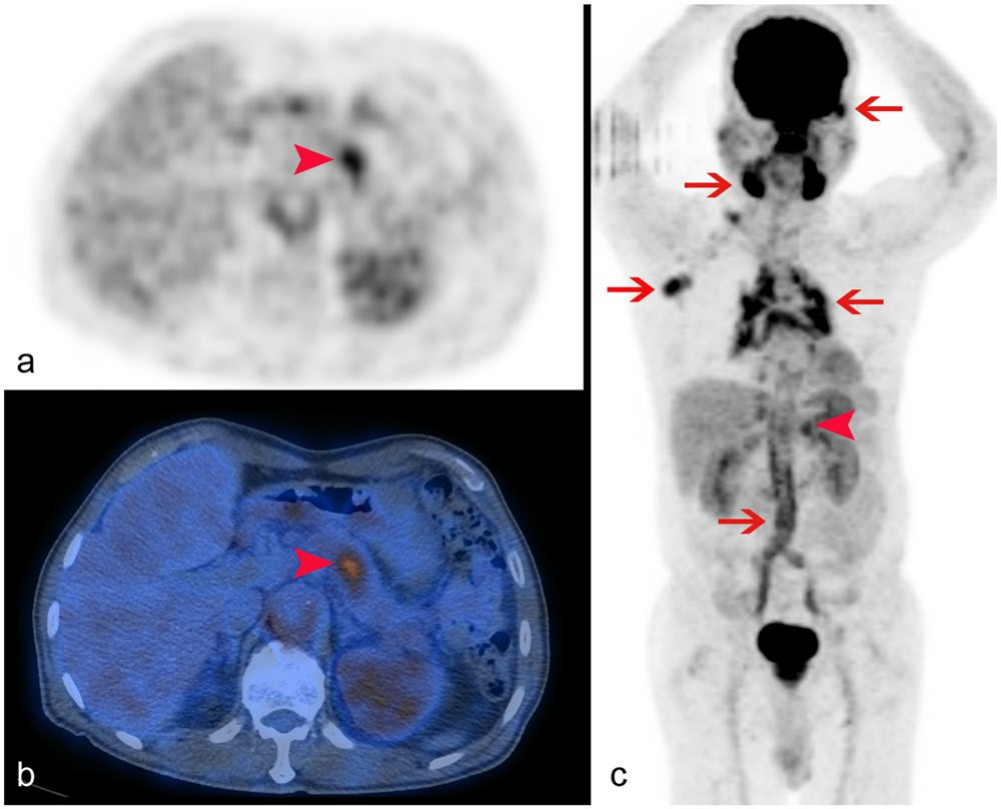
Autoimmune pancreatitis, focal type. Axial (**a**) and PET/CT fused (**b**) images showed a localized focus of intense hypermetabolism at the pancreatic body (arrowhead). The maximum intensity projection image (**c**) revealed more than two foci of extrapancreatic uptake, including the submandibular and left lacrimal glands, axillary and mediastinal nodes, and aorta to common iliac arteries (arrows). The primary pancreatic tumor exhibited a SUVmax of 5.1, and a high gray-level zone emphasis value of 125.4.

**Figure 3:**
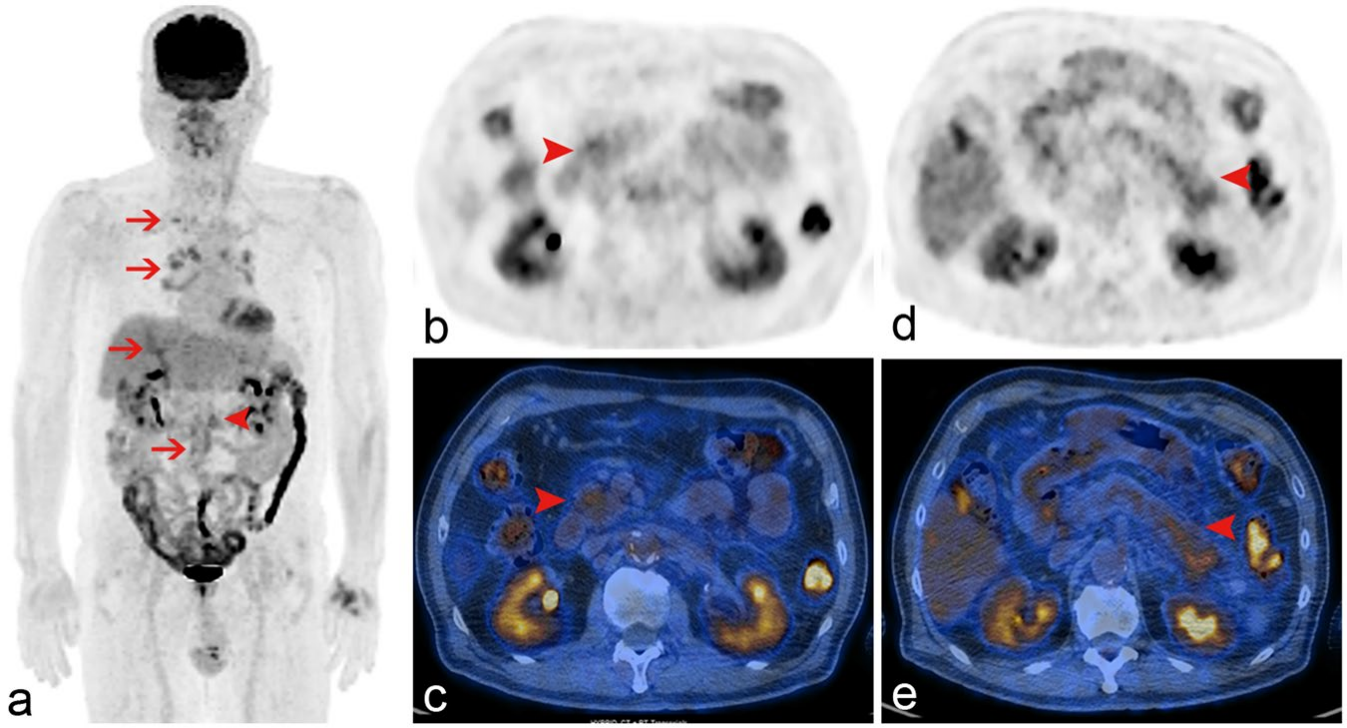
Autoimmune pancreatitis, diffuse type. The maximum intensity projection image (**a**) revealed more than two foci of extrapancreatic uptake, including left supraclavicular and mediastinal nodes, bile ducts, and abdominal aorta (arrows). Axial (**b**,**d**) and PET/CT fused (**c**,**d**) images showed patchy diffuse mild to moderate hypermetabolism at pancreatic head to tail (arrowhead). The primary pancreatic tumor exhibited a SUVmax of 6.3 and a low high gray-level zone emphasis value of 94.3.

**Figure 4:**
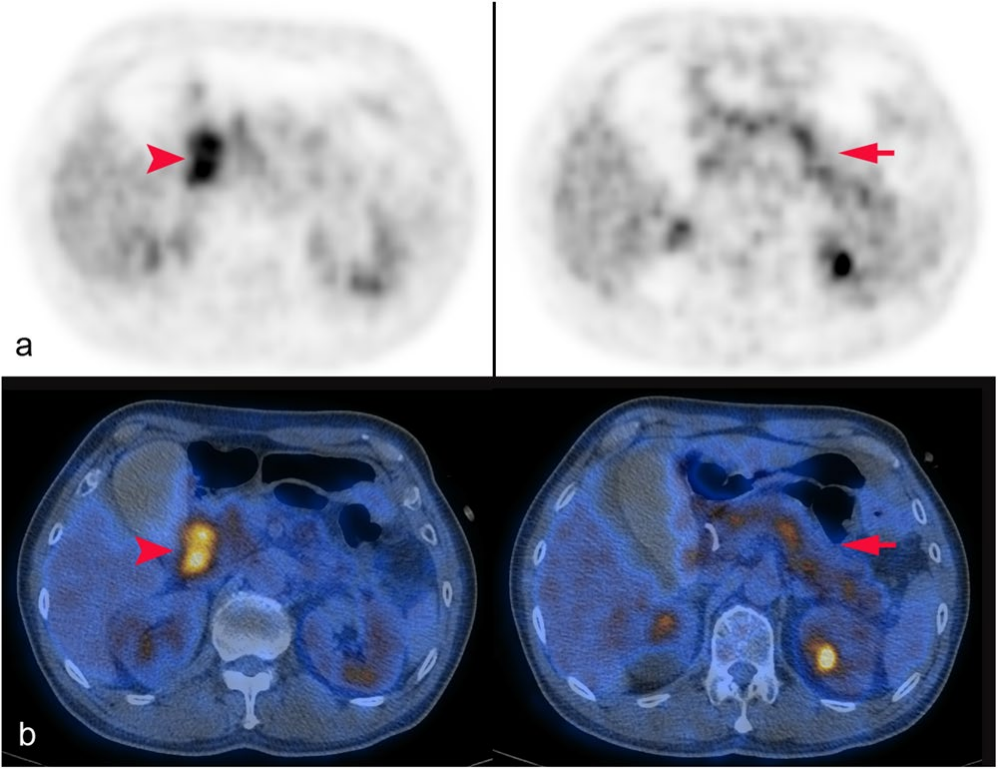
Pancreatic cancer. Axial PET (**a**) and PET/CT fused (**b**) images showed intense hypermetabolic areas from pancreatic head to tail (diffuse morphology, arrow), and the most intense focal area at periampullary region (arrowhead). No definite extrapancreatic lesion was found. The pancreatic tumor showed a SUVmax of 8.4, and a high gray-level zone emphasis value of 201.3. The patient underwent Whipple’s surgery confirming the diagnosis of pancreatic cancer. The disease was complicated with ischemic bowel disease and multi-organ failure developed, and the patient died despite intensive care support.

Further logistic regression analysis yielded diffuse pancreatic tumor morphology, more than two extrapancreatic sites of FDG uptake, primary tumor exhibiting a lower maximum SUV, and lower high gray-level zone emphasis (HGLZE) value most optimal for predicting AIP (Figs 2–4, and Table from Supplementary data). The prediction model formulated from the combination of these four parameters showed an AUC of 0.95 (*P* < 0.0001, Table 3 and Fig. 1 from Supplementary data). The sensitivity, specificity, PPV, NPV, and accuracy of the formulated prediction model were 90.6% (95% CI, 80.7–96.5%), 84.0% (95% CI, 70.9–92.8%), 87.9% (95% CI, 79.3–93.2%), 87.5% (95% CI, 76.4–93.8%), and 87.7% (95% CI, 80.3–93.1%), respectively, for determining AIP from PC in the cohort of 114 patients included in the study (Table 4).

**Table 3:** ROC Analysis for Differentiation Between Autoimmune Pancreatitis and Pancreatic Cancer (*n* = 114).

Parameter	Optimal threshold for diagnosing pancreatic cancer	Odds Ratio	AUC (CI)	*P* value
Number of extrapancreatic lesions	<2	2.9	0.60 (0.50–0.70)	0.009
Pancreatic pattern	localized	10.2	0.73 (0.63–0.81)	<0.0001
SUV max	>6.8	2.2	0.74 (0.65–0.82)	<0.0001
High gray-level zone emphasis	>131.3	1.1	0.77 (0.68–0.85)	<0.0001
Combined			0.95 (0.88–0.98)	<0.0001

AUC: area under the ROC curve, CI: confidence intervals, ROC: Receiver-operating-characteristic curve.

**Table 4:** Differentiating Pancreatic Cancer from Autoimmune Pancreatitis in the 114 Patients Using FDG-PET Derived Parameters.

FDG PET/CT	Pancreatic Cancer		Total (*n*)
	Present (*n*)	Absent (*n*)	
Positive (*n*)	58	8	66
Negative (*n*)	6	42	48
Total (*n*)	64	50	114

### Pancreatic Malignancies Found in Patients Suspected of AIP

Pancreatic malignancies were detected in 3 patients with suspected AIP using FDG PET/CT prior to initiation of steroid therapy. Two subjects have concomitant proven IgG4-RD which both salivary glands and mediastinal lymph nodes involvement were seen in their PET/CT scans (Fig. 2 from Supplementary data). Their pancreatic lesions were of localized morphology with SUVmax and an HGLZE value greater than 5 and 140, respectively. Also, in another patient with suspected focal type AIP, pancreatic tumor HGLZE value was high (255.2) despite a SUVmax of less than 5, and only one site of extrapancreatic uptake (mediastinal lymphadenitis) was seen in FDG PET/CT. This patient later underwent surgery confirming the presence of a pancreatic tail adenocarcinoma.

## Discussion

Therapy for AIP and PC is markedly different. These two entities are at times hard to distinguish due to overlapping clinical features, and difficulty in obtaining adequate biopsy specimens for diagnosis. Herein, this study aims to determine whether addition of FDG PET/CT in the clinical algorithm can provide clinical useful information in patients suspect of AIP before therapy.

The present study showed that (1) AIP usually exhibits diffuse pancreatic uptake, lower SUVmax and HGLZE values than PC; (2) More than 80% of AIP patients had at least one site of extrapancreatic inflammation, and half of them showed least 2 sites of involvement; (3) The location of extrapancreatic lesions also differed between AIP and PC, with preference of salivary glands and mediastinal lymph nodes involvement in AIP. Moreover, lacrimal glands, axillary lymph nodes, vessel walls, and the pituitary gland were only observed in our AIP cohort; (4) PET successfully detected pancreatic malignancy in 3 patients initially suspect of AIP before steroid therapy, 2 of which also proved to have systemic inflammatory foci owing to underlying IgG4-RD. Hence, FDG PET/CT showed sensitivity, specificity, accuracy of 90.6%, 84.0%, and 87.7% in differentiating PC from AIP.

Extrapancreatic manifestation of AIP has been described since Yoshida *et al*.^8^ reported the first case of type I AIP in 1995. Subsequently, an elevated serum level of IgG4 has been linked to the disease, and the disease considered IgG4-related autoimmune disease (IgG4-RD) owing to its systemic multi-organ involvement^3,9^. Prompt diagnosis of AIP is challenging, and can be hard to distinguish from PC in patients who present with obstructive jaundice, mild abdominal discomfort, and weight loss^10–12^. Abdominal CT or magnetic resonance imaging does not provide information specific for AIP, especially in patients with atypical imaging features such as a low-density mass in CT, pancreatic ductal dilatation, or distal atrophy^7^, which can mimic various neoplastic processes. In addition, no standard laboratory parameter, including serum IgG4 concentration, is reliable for diagnosing AIP or illustrating the extent of IgG4-RD. Not all patients with AIP present with an elevated serum IgG4 level, nor is a rising level indicative of the disease^13^. Patients with respiratory, biliary, rheumatic, and liver disease, and even those with PC can have an elevated serum IgG4 level^14,15^. Moreover, an increased incidence of malignancy in patients with AIP or IgG4-RD pancreatitis has been reported^16–18^. Accurate diagnosis is of utmost importance since only surgical resection offers curative treatment for PC, whereas corticosteroid or rituximab treatment are given for patients with AIP.

Published studies have described the usefulness of FDG PET or FDG PET/CT for determining the prevalence and distribution of extrapancreatic lesions in AIP, and systemic manifestations of IgG4-RD^19–23^. PET has the added advantages of whole-body screening, the ability to highlight unsuspected lesions involving critical organs such as the pericardium, kidneys, aorta, proximal biliary structures, and retroperitoneum, and can help determine the extent of disease^23,24^. More than half of AIP patients fail to achieve sustained remission after initial corticosteroid therapy^13^, and it has been shown that rituximab might be an effective treatment^25,26^. Therefore, whole-body screening provided by FDG PET/CT can be used to assess response to therapy. Consistent with previous work, the current study showed more frequent salivary glands and mediastinal lymph nodes involvement in AIP^21,27^. In addition, lacrimal glands, axillary lymph nodes, vessel walls, and pituitary gland activities were only seen AIP patients compared to PC. Similar findings were also reported by the UK, Japanese, Korean, Chinese, and Taiwanese researchers^28–31^.

At present, no studies have compared the texture features between AIP and PC. Spatial variation in intensity represents tumor heterogeneity in PET images, which can be analyzed using texture analysis. Tumor heterogeneity reflects regional variation in cellular proliferation, hypoxia, necrosis, metabolic activity, and vascular structure^32^. Theoretically, heterogeneity should differ between PC and AIP. Our study suggests that in addition to visual analysis and volumetric quantification methods, addition of the higher-dimensional heterogeneity feature, HGLZE, can aid in differentiate AIP from PC. HGLZE reflects spatial variation of voxel-gray-scale intensity within an image. It emphasizes the “discrete” high-intensity regions within a tumor regardless the tumor size itself. Therefore, a tumor with multiple scattered focal hypermetabolic regions will have a higher HGLZE value than a tumor of homogeneous uptake or containing only a single focal hypermetabolic region. Images of selected cases are presented in Figs 2–4, and Supplementary Data. Interestingly, 3 of 53 patients initially suspected of AIP were found to harbor PC and their HGLZE values were all above 140, regardless of their pancreatic tumor SUVmax or morphology.

Several limitations of this study that could limit the conclusions should be considered. The major limitation is the retrospective nature with relatively small number of patients diagnosed with AIP. Second, more than 90% of the AIP patients presented with elevated serum IgG4 level, hence these patients were probably of a more severe disease phenotype^13^. Nevertheless, these patients were also more likely to require prompt treatment because of their disease status, and excluding malignancy is of ultimate importance. We used the organ-specific criteria for AIP for all patients in the study such that the selection criteria were consistent, instead of the newer comprehensive criteria proposed by Umehara *et al*. in 2012. Nonetheless, all of our patients fit the diagnosis of AIP according the latest 2011 International Consensus Diagnostic Criteria at the time of analysis. Also, it was not until 2011 that all enrolled AIP patients were checked for serum cancer antigen 19-9 (CA 19-9) level, and IgG4 titers were analyzed for all PC patients. Since it has been shown that not all AIP patients exhibit an increased serum IgG4 level, nor is an elevated CA 19-9 level found in all PC patients, we believe the study provides more evidence for the role of FDG PET/CT in AIP. In addition, we did not evaluate PET texture features in other more common forms of pancreatitis due to logistic reasons (not fitting the criteria for performing FDG PET/CT in routine clinical practice). Histological confirmation of extrapancreatic inflammatory foci detected by FDG PET/CT was not possible for all patients with AIP due to ethical reasons, particularly in locations where tissue is difficult to obtain (such as retroperitoneum or pituitary gland). Nonetheless, most AIP patients responded well to the initial steroid therapy and their follow-up radiological studies showed complete or partial regression of these extrapancreatic inflammatory lesions. Moreover, these inflammatory lesions detected by FDG PET/CT were observed on usual locations known to be involved by IgG4-RD. It is well known that a combination of clinical, biochemical, and CT imaging features can differentiate benign from malignant pancreatic masses in >90% of cases. In the remaining 10% of cases, albeit high sensitivity of FDG PET/CT, its specificity in differentiating AIP from PC remains suboptimal, thus it is better to have histopathology proof of the pancreatic lesion. Endoscopic ultrasonography-guided fine needle aspiration (EUS-FNA) offers a relative easy way of tissue sampling. However, the sensitivity of the positive IgG4 immunostaining in biopsy specimens depends on the disease stage and activity, and also the amount of specimens. Although the advance of EUS-FNA needles can improve the quality and quantity of the pancreatic histological samples, these new generation of needles are not available in our hospital until last year, and not used in our study cohort. In addition, the diagnosis of AIP relies on not only the histopathology, but also other cardinal features. Therefore a conventional whole body FDG PET/CT can provide useful information in delineating the extent of disease involvement outside the pancreas, which may help to confirm the diagnosis of AIP when biopsy results are inconclusive. Further prospective studies enrolling a larger number of patients, with tissue proof, are needed to validate and strengthen the study results.

In conclusion, the combination of FDG-PET parameters and texture analysis may provide additional clinical useful information in patients with suspected AIP prior to steroid therapy. Extrapancreatic involvement found by FDG PET/CT can be helpful in supporting the diagnosis of AIP when the pancreatic findings alone are indeterminate. This may be especially useful in clinically difficult cases where coexisting malignancy is highly suspected, but biopsy is inconclusive.

## Methods

### Patient Selection and Study Criteria

From September 2008 to July 2016, 53 consecutive patients (Table 1) with suspected AIP according to the 2008 Asian Diagnostic Criteria^13^ were enrolled in the present study prior initiation of steroid therapy. Patients with another malignancy still under treatment, received abdominal surgery in recent 3 months, pregnant, lactation or uncontrolled medical disease other than cancer were excluded from the study. Their FDG PET/CT images were compared with those of 61 patients later pathologically proven have PC during the same study period. Each patient underwent a conventional diagnostic work-up including a review of systems, physical examination, and an abdominal CT or magnetic resonance imaging. Eighty-five patients (75.2%) received EUS and 33 (29.2%) endoscopic retrograde cholangiopancreatography. All conventional imaging studies and procedures were performed within 2 weeks of PET/CT. Patients were followed at the outpatient clinic for at least 6 months after the PET/CT or until death (median, 32.5 months; range, 6-82.9 months).

The National Taiwan University Hospital Ethics Committee approved this clinical study. All participants underwent PET/CT after providing written informed consent and the study was performed in accordance with relevant guidelines and regulations. All patients consent to publish relevant information/images used in the present study.

### FDG PET/CT Imaging

All patients fasted for at least 4 hours to maintain serum glucose concentrations below 180 mg/dL before intravenous injection with 370 MBq (10 mCi) of FDG. PET/CT image acquisition at 45-min (early-phase) and 2-hour post-injection (delayed-phase) using the same PET/CT scanner was performed, and followed the same protocol as described in our previous study^14^.

### PET/CT Data Analysis

Two nuclear medicine physicians (*MF Cheng* and *YW Wu*) with >10 years of clinical experience, unaware of the results of other diagnostic tests, histology, and final diagnosis, reviewed the images independently using the built-in software (eNTEGRA, GE Medical Systems, WI). Diverging interpretations were resolved by consensus.

Increased FDG activity in the pancreas greater than the surrounding background activity, and not associated with normal structures or artifacts in the FDG PET transaxial slices were analyzed visually for uptake morphology. A pancreatic lesion with diffuse morphology was defined as uptake in more than two or contiguous segments, otherwise the lesion was characterized as having a localized morphology^15^. A pancreatic lesion was classified as AIP if the lesion showed patchy FDG distribution in the pancreas without focal intense activity, and more than 2 sites of extrapancreatic FDG uptake were found. Otherwise, the pancreatic lesion was classified as malignant. The number of extrapancreatic FDG-avid lesions was counted for each patient. Patients were grouped into two categories: those with two or more and those with less than two sites of extrapancreatic FDG-avid lesions.

For semiquantitative evaluation, all foci with abnormally increased FDG uptake were evaluated by placing a volume of interest (VOI) in the suspected pancreatic lesion seen in the co-registered CT images. The standardized uptake values (SUVs) normalized to body weight (SUV = tissue concentration. injected dose^−1^ body weight^−1^) were acquired using the attenuation corrected images. For quantitative analysis, metabolic volume (MTV) was automatically selected in the axial PET images with maximum SUV (SUVmax) ≥ 2.5 as the primary pancreatic tumor, as identified by the nuclear medicine physicians. Adjustments were made if non-tumor areas were incorrectly included within the VOI. Only lesions with a MTV > 5 mL (mean 41.1 ± 3.9 mL, range 8.2-295.2 mL) were included in the analysis to avoid the partial volume effect^16^.

In addition to lesion SUVmax, MTV, and SUVmax delayed/early ratio (SUVR), the total lesion glycolysis (TLG) of the lesion were also calculated. For all tumors, the FDG PET data were quantized into 32 bins, followed by textural analysis via first-order and high-order primary tumor texture features, as described in the Supplementary Data. A total of 19 PET texture indices commonly used in medical imaging research were included in the analysis^17^ (Supplementary data).

### Statistical Analysis

All aforementioned parameters were examined for their ability to distinguishing AIP from PC by comparing one morphological parameter and outcome (AIP or PC) one at a time. Parameters with a significant relationship (*P* < 0.01) were then included in a backward stepwise logistic regression analysis. A prediction model was formulated using significant parameters from the above backward stepwise logistic regression analysis. Only those parameters with predictive probability of 0.35 or higher were included in the final prediction model. The corresponding area under the receiver operating characteristic curve (AUC) was reported. The resulting prediction model was examined to determine sensitivity, specificity, positive predictive value (PPV), and negative predictive value (NPV) in differentiating PC from AIP.

Numerical data were expressed as mean ± standard deviation (SD). Quantitative parameters were compared using two-tailed Student *t* test. Values of *P* < 0.05 were considered statistically significant. All analyses were conducted with the JMP®, version 5 statistical software package (SAS Institute Inc., Cary, NC, USA).

### Data availability

All data generated or analyzed during this study are included in this article (and its Supplementary Information files).

## Electronic supplementary material

Supplementary Data

Supplementary Figure1

Supplementary Figure2
