# Supplementary material for: Clinical Utility of FDG PET/CT in Patients with Autoimmune Pancreatitis: a Case-Control Study
Source: Sci Rep. 2018 Feb 26;8:3651. doi: 10.1038/s41598-018-21996-5 (PMC5827761; doi:10.1038/s41598-018-21996-5)
Supplement: Supplementary file 1 — Supplementary Data [file 41598_2018_21996_MOESM1_ESM.doc]

**Clinical Utility of FDG PET/CT in Patients with Autoimmune Pancreatitis: a Case-Control Study**

Mei-Fang Cheng^1,2^, MD; Yue Leon Guo^2,3^, MD, PhD; Ruoh-Fang Yen^1^, MD, PhD; Yi-Chieh Chen^1^, MSc; Chi-Lun Ko^1^, MD; Yu-Wen Tien^5^, MD, PhD; Wei-Chih Liao^8^, MD, PhD; Chia-Ju Liu^1^, MD; Yen-Wen Wu,^1,6,7*^, MD, PhD; Hsiu-Po Wang^8*^, MD.

*These authors contributed equally to the study.

^1^Department of Nuclear Medicine, ^3^Environmental and Occupational Medicine, ^5^Surgery, and ^8^Internal Medicine, National Taiwan University College of Medicine and Hospital, Taipei City, Taiwan

^2^Institute of Occupational Medicine and Industrial Hygiene, National Taiwan University, Taipei, Taiwan

^3^Department of Nuclear Medicine, National Taiwan University Hospital, Yun-Lin Branch, Yun-Lin County, Taiwan;

^6^Department of Nuclear Medicine and Cardiovascular Medical Center (Cardiology), Far Eastern Memorial Hospital, New Taipei City, Taiwan

^7^National Yang-Ming University School of Medicine, Taipei, Taiwan;

**Textural Feature Extraction**

The FDG PET images were quantitatively analyzed by using our in-house software written in C++ (Visual Studio 2015, Microsoft Corporation). Images were normalized and discretized into 32 levels according to the maximum and minimum SUV within the VOI. All textural features were evaluated in a three-dimensional 26-connected scheme. A total of 19 indices were generated from the three matrices.

The gray-level co-occurrence matrix (GLCM) was generated by calculating the probability of the co-occurrence of two neighboring voxels (distance = 1) in all 13 possible directions (under the three-dimensional 26-connected scheme). Six textural indices were generated by the following formulae:


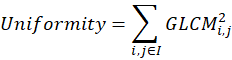


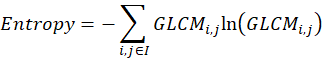


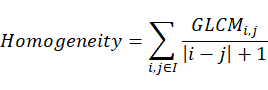


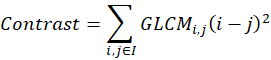


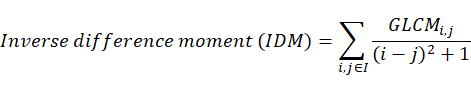


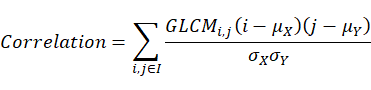


Where,
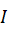
 represents the set of possible intensity levels,
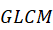
 represents the normalized co-occurrence matrix,
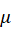
 and
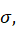
 respectively, represent the mean and standard deviation of weighted intensity levels along rows and columns of the matrix. We used the root-mean-square method to summarize the textural indexes in each of the 13 possible directions.

The neighborhood gray-tone difference matrix (NGTDM) was created by calculation of the sum of the absolute difference between the voxels of a given intensity and their 26-connected neighbors. Five textural indexes were generated from the difference matrix:


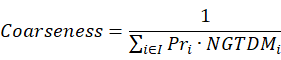


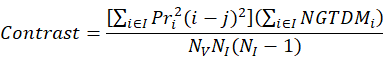


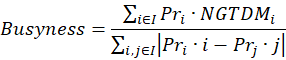


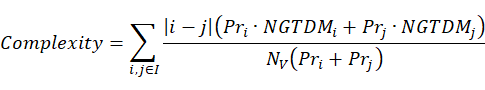


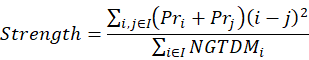


Where
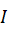
 represents the set of possible intensity levels,
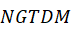
 represents the neighborhood gray-tone difference matrix,
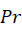
 represents the probability of occurrence of voxels with a given intensity,
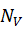
 represents the number of voxels, and
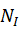
 represents the number of intensity levels.

Finally, the three-dimensional gray-level size-zone matrix (SZM) was generated by using connected component analysis. The occurrence probabilities of zones with given sizes and given intensity levels were calculated. A total of eight textural indexes were calculated:


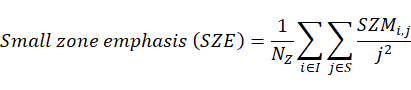


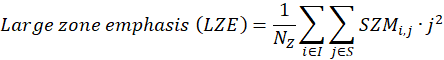


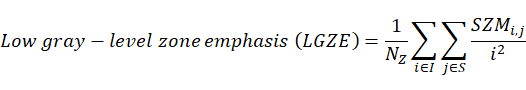


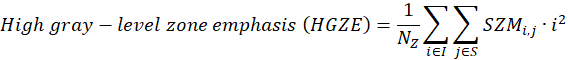


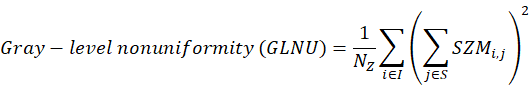


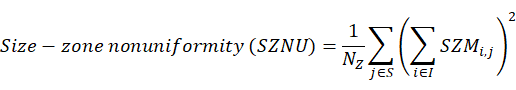


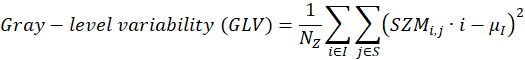


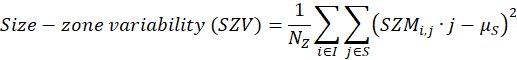


Where
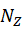
 is the total number of zones,
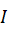
 represents the set of possible intensity levels,
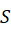
 represents the set of possible zone sizes,
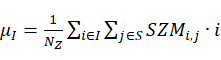
, and
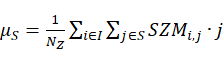
.

**SUPPLEMENTARY TABLE. Results of Univariate and Multivariate Analyses of PET Parameters in 114 Patients**

| Analysis Type and Parameter | Pancreatic cancer (n=64) | Autoimmune Pancreatitis (n=50) | Univariate Analysis | Multivariate Analysis |
| --- | --- | --- | --- | --- |
|  | mean (SE) | mean (SE) | *P*-value | |
| SUVmax | 7.8 (0.5) | 5.0 (0.2) | <0.0001* | 0.0002* |
| SUVmean | 3.8 (0.1) | 2.8 (0.1) | <0.0001* | NS |
| SUVratio | 1.2 (0.03) | 1.1 (0.03) | NS | NS |
| Metabolic tumor volume (mL) | 34.4 (5.6) | 49.1 (5.3) | NS | NS |
| Total lesion glycolysis | 141.0 (24.3) | 142.1 (19.4) | NS | NS |
| Gray-Level Co-occurrence Matrix | | | | |
| Uniformity | 0.01 (0.002) | 0.02 (0.004) | NS | NS |
| Entropy | 4.78 (0.08) | 4.82 (0.12) | NS | NS |
| Homogeneity | 0.28 (0.01) | 0.29 (0.01) | NS | NS |
| Contrast | 59.6 (3.4) | 54.7 (4.7) | NS | NS |
| IDM | 0.19 (0.01) | 0.21 (0.01) | NS | NS |
| Correlation | 0.13 (0.001) | 0.10 (0.01) | NS | NS |
| Neighborhood Gray-Tone Difference Matrix | | | | |
| Coarseness | 0.02 (0.002) | 0.01 (0.001) | 0.0023* | NS |
| Contrast | 0.45 (0.04) | 0.38 (0.05) | NS | NS |
| Busyness | 0.45 (0.04) | 1.02 (0.29) | <0.0001* | NS |
| Complexity | 1412.0 (57.8) | 1417.0 (65.5) | NS | NS |
| Strength | 9.0 (0.6) | 6.2 (0.6) | 0.0012* | NS |
| Gray-Level Size-Zone Matrix | | | | |
| Small zone emphasis | 0.70 (0.01) | 0.70 (0.01) | NS | NS |
| Large zone emphasis | 77.3 (67.1) | 143.0 (130.0) | NS | NS |
| Low gray-level zone emphasis | 0.09 (0.01) | 0.12 (0.001) | 0.0008* | NS |
| High gray-level zone emphasis | 172.8 (6.7) | 137.4 (7.6) | 0.0006* | 0.0003* |
| Gray-level nonuniformity | 11.5 (1.8) | 18.8 (1.5) | 0.0029* | NS |
| Size-zone nonuniformity | 87.9 (9.5) | 137.5 (10.7) | 0.0007* | NS |
| Gray-level variability | 950.0 (224.1) | 819.5 (101.9) | NS | NS |
| Size-zone variability | 392.6 (367.8) | 2804.3 (2772.5) | NS | NS |
| Pancreatic morphology | | | | |
| Diffuse, *n* (%) | 3 (4.9) | 26 (52.0) | <0.0001* | 0.0001* |
| Localized, *n* (%) | 58 (95.1) | 24 (48.0) |  |  |
| Number of extrapancreatic lesions | | | | |
| <2, *n* (%) | 48 (75.0) | 25 (50.0) | 0.013* | 0.007* |
| ≥2, *n* (%) | 16 (25.0) | 25 (50.0) |  |  |

NS: not significant, SUV: standard uptake value, IDM: inverse difference moment.

Note: data in parentheses are standard errors (SE) unless otherwise stated.

*P* < 0.01 in the univariate analysis and *P* < 0.05 in the multivariate analysis indicate significant differences (*).

**SUPPLEMENTARY FIGURE LEGENDS**

**SUPPLEMENTARY FIGURE 1.** Receiver operating characteristic **(**ROC) curves of the four PET parameters used in the prediction model (a), and the combined prediction model (b), for differentiation between autoimmune pancreatitis and pancreatic cancer. AUC: area under curve; extra: number of extrapancreatic lesion sites; SUVmax-e: early-phase maximum standardized uptake value.

**SUPPLEMENTARY FIGURE 2.** Pancreatic malignancy in a patient with histopathologically proven IgG4-RD prior to initiation of steroid therapy. Axial PET (a), fused PET/CT (b), and the maximum intensity projection (c) images revealed a localized intense hypermetabolic area at the pancreatic body (arrowhead) with a SUVmax of 7.5, and a high-grey level zone value of 148.8. Bilateral salivary glands and mediastinal nodes showed intense uptake. Subsequent surgery revealed a stage IIIB pancreatic adenocarcinoma.
