# Supplementary figures and images for: Clinical Utility of FDG PET/CT in Patients with Autoimmune Pancreatitis: a Case-Control Study
Source: Sci Rep. 2018 Feb 26;8:3651. doi: 10.1038/s41598-018-21996-5 (PMC5827761; doi:10.1038/s41598-018-21996-5)

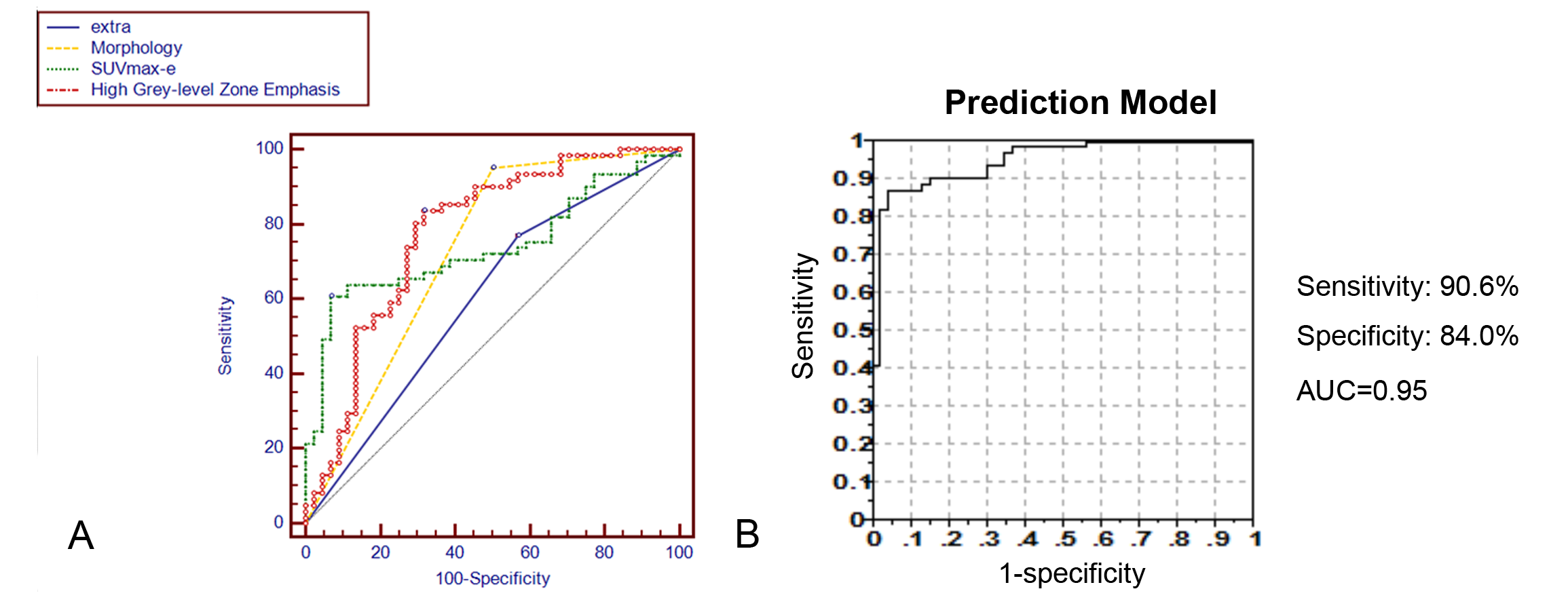

Supplement: Supplementary file 2 — Supplementary Figure1 [file 41598_2018_21996_MOESM2_ESM.tif]

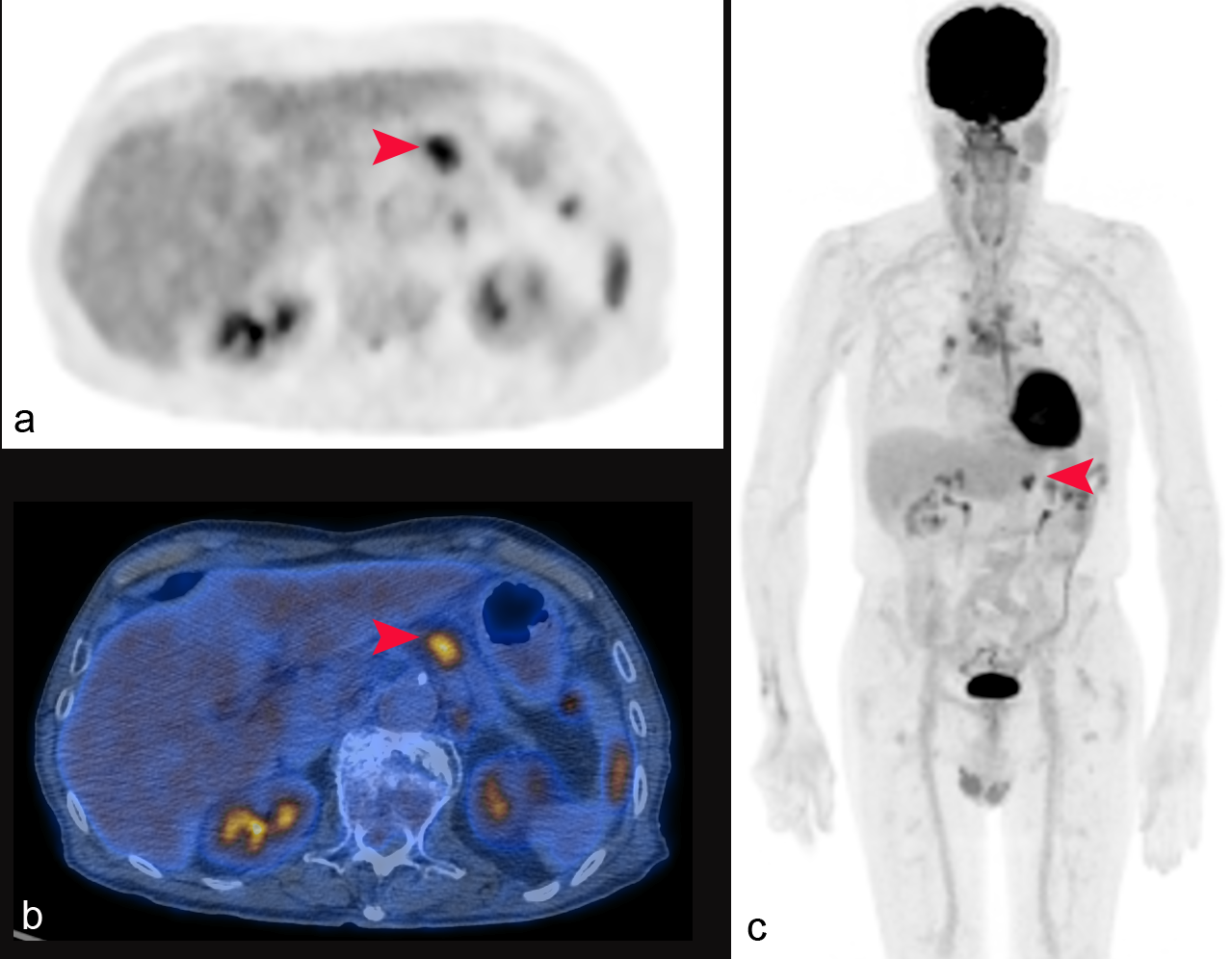

Supplement: Supplementary file 3 — Supplementary Figure2 [file 41598_2018_21996_MOESM3_ESM.tif]
